# Fabrication of 3D printed mutable drug delivery devices: a comparative study of volumetric and digital light processing printing

**DOI:** 10.1007/s13346-024-01697-5

**Published:** 2024-08-23

**Authors:** Ye Chan Oh, Jun Jie Ong, Haya Alfassam, Eduardo Díaz-Torres, Alvaro Goyanes, Gareth R. Williams, Abdul W. Basit

**Affiliations:** 1 Department of Pharmaceutics, UCL School of Pharmacy, University College London, 29-39 Brunswick Square, London, WC1N 1AX UK; 2 Advanced Diagnostics and Therapeutics Institute, King Abdulaziz City for Science and Technology (KACST), Health Sector, Riyadh, 11442 Saudi Arabia; 3 Instituto Universitario de Enfermedades Tropicales y Salud Pública de Canarias, Universidad de La Laguna, La Laguna, 38203 Spain; 4 Programa de Doctorado en Ciencias Médicas y Farmacéuticas, Desarrollo y Calidad de Vida, Universidad de La Laguna, La Laguna (Tenerife), 38200 Spain; 5 Departamento Ingeniería Química y Tecnología Farmacéutica, Universidad de La Laguna, La Laguna, 38200 Spain; 6 Departamento de Farmacología, Farmacia y Tecnología Farmacéutica, Facultad de Farmacia, Instituto de Materiales (iMATUS) and Health Research Institute of Santiago de Compostela (IDIS), Universidade de Santiago de Compostela, Santiago de Compostela, 15782 Spain; 7 FabRx Ltd., Henwood House, Henwood, Ashford, Kent, England, TN24 8DH UK

**Keywords:** Additive manufacturing of drug products, 3D printing of pharmaceuticals and medications, Gastro-retentive drug delivery systems, Vat photopolymerization, Oral formulations and medicines

## Abstract

Mutable devices and dosage forms have the capacity to dynamically transform dimensionally, morphologically and mechanically upon exposure to non-mechanical external triggers. By leveraging these controllable transformations, these systems can be used as minimally invasive alternatives to implants and residence devices, foregoing the need for complex surgeries or endoscopies. 4D printing, the fabrication of 3D-printed structures that evolve their shape, properties, or functionality in response to stimuli over time, allows the production of such devices. This study explores the potential of volumetric printing, a novel vat photopolymerisation technology capable of ultra-rapid printing speeds, by comparing its performance against established digital light processing (DLP) printing in fabricating hydrogel-based drug-eluting devices. Six hydrogel formulations consisting of 2-(acryloyloxy)ethyl]trimethylammonium chloride solution, lithium phenyl-2,4,6-trimethylbenzoylphosphinate, varying molecular weights of the crosslinking monomer, poly(ethylene glycol) diacrylate, and paracetamol as a model drug were prepared for both vat photopolymerisation technologies. Comprehensive studies were conducted to investigate the swelling and water sorption profiles, drug release kinetics, and physicochemical properties of each formulation. Expandable drug-eluting 4D devices were successfully fabricated within 7.5 s using volumetric printing and were shown to display equivalent drug release kinetics to prints created using DLP printing, demonstrating drug release, swelling, and water sorption properties equivalent to or better than those of DLP-printed devices. The reported findings shed light on the advantages and limitations of each technology for creating these dynamic drug delivery systems and provides a direct comparison between the two technologies, while highlighting the promising potential of volumetric printing and further expanding the growing repertoire of pharmaceutical printing.

**Graphical Abstract:**

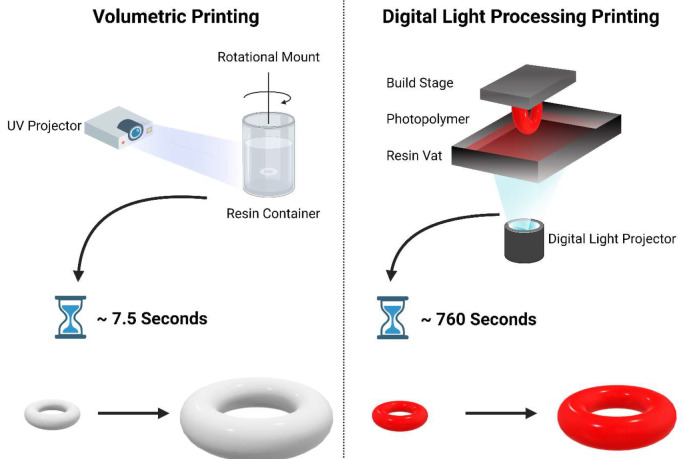

**Supplementary Information:**

The online version contains supplementary material available at 10.1007/s13346-024-01697-5.

## Introduction

Mutable devices and dosage forms are capable of self-transforming their shape, property or functionality when exposed to non-mechanical external stimulus, such as heat, pH, light, moisture, electric or magnetic fields, or ion composition [1]. The capacity to induce dimensional, morphological and mechanical changes upon contact with non-mechanical external triggers is especially considered a promising characteristic for the fabrication of minimally invasive implants or residence devices in targeted areas of the body [2]. By utilizing temporary, smaller, shapes for easier administration (e.g., via catheters, syringes, or even oral ingestion), these devices can then undergo a controlled transformation upon reaching their target, ensuring sustained drug release within the targeted area [3]. While established residence devices such as intragastric balloons for obesity and stents show clinical success, many rely on procedures like endoscopies, surgeries, and anaesthesia, underscoring the demand for minimally invasive alternatives [4, 5].

Additive Manufacturing (AM), or three-dimensional printing (3DP), is an innovative technology able to generate 3D objects from computer aided design (CAD) models, generally through a layer-by-layer process. 3DP has been transformative in the healthcare industry, as its ability to fabricate complex geometric structures [6] with high drug loading and modifiable properties has the potential to address limitations faced by traditional manufacturing methods [7]. The various 3D printing technologies have been classified into seven broad categories by the American Society for Testing and Materials (ASTM) International: material extrusion, material jetting, directed energy deposition, sheet lamination, vat photopolymerisation, powder bed fusion, and binder jetting [8]. These 3DP technologies each possess distinct characteristics and involve different feedstock materials that define their potential uses and capabilities. Vat photopolymerisation-based technologies offer the highest printing resolution of all the printing technologies, being able to print models of micron-scale with high dimensional accuracy and printing precision [9, 10]. This printing method involves selective solidification of liquid photopolymerizable materials through exposure to light. Digital light processing (DLP) printing, categorised under vat photopolymerization, utilises *projection* light to polymerise materials to fabricate three-dimensional objects. This technology has been extensively used in the field of pharmaceutics to manufacture various drug dosage forms, medical devices, and drug delivery systems. However, these more established vat photopolymerisation technologies are limited by relatively slow printing speeds.

Volumetric printing is a novel vat photopolymerisation technology capable of ultra rapid fabrication of high-resolution 3D objects. Volumetric printing’s rapid printing speed is achieved by synchronously illuminating ultraviolet (UV) light at the photopolymerisable liquid from multiple angles, fabricating the object in multiple directions simultaneously [11]. As such, volumetric printing does not employ a layer-by-layer process: instead, the structure is directly printed in three-dimensions in a layerless fashion, foregoing the need for support structures commonly seen in other layer-by-layer technologies [12]. The utilisation of volumetric printing in pharmaceutics has been explored, with drug loaded torus printlets able to be printed in seconds [13], and the ability to simultaneously print multiple drug-loaded printlets [14] for even faster fabrication.

The adaptation of stimuli responsive materials—namely, shape memory polymers (SMPs), stimuli-responsive hydrogels, or liquid crystal (LC) polymers—in 3D printing has allowed for the printing of stimuli responsive, mutable, structures with tuneable morphologies [2, 3]. The term 4D printing (4DP) was introduced to encompass all 3DP technologies that produce objects capable of having programmable time-dependent alterations [15]. The increase in utilisation of conventional 3D printing technologies for 4D printing has been seen in a myriad of fields. Notable applications of 4D printing include heat-activated flexible temperature sensors [16], cardiac patches [17], drug eluting stents and implants [18], scaffolding for tissue regeneration [2], hydrogel-based wound dressings for simultaneous monitoring and drug delivery [19], biocompatible smart scaffolds [20], and self-folding hydrogel scaffolds [21]. Volumetric printing has shown merit in pharmaceutical sciences for preparing simple oral dosage forms at ultra-rapid speeds; however, other potential applications and the versatility of this technology have largely been unexplored, particularly its ability to create mutable drug delivery devices and dosage forms.

This study focuses on two vat photopolymerisation technologies: DLP (digital light processing) and volumetric printing, comparing their efficacy and suitability for fabricating mutable hydrogel-based drug-eluting devices with potential application as gastro-retentive drug delivery systems. Hydrogels, due to their inherent mechanical properties, pose minimal risk of tissue penetration, making them well-suited for gastro-residence purposes [22]. These mutable drug-eluting devices were successfully fabricated using both vat photopolymerisation technologies, and studies were conducted to comparatively investigate their swelling characteristics, drug release kinetics, and physicochemical properties. This comparative study investigates the potential of volumetric printing in creating these dynamic drug-eluting devices, evaluating its performance against the conventional DLP approach.

## Materials & methods

### Materials

2-(Acryloyloxy)ethyl]trimethylammonium chloride solution (TMAEA) (80 wt% in water), polyethylene glycol diacrylate (PEGDA) (250 g/mol, 575 g/mol, 700 g/mol), and lithium phenyl-2,4,6-trimethylbenzoylphosphinate (LAP, MW 294.21 g/mol, ≥ 95%) were purchased from Sigma-Aldrich (Gillingham, UK). Paracetamol and solvents (water; methanol) for HPLC analysis were of HPLC grade and sourced from Sigma-Aldrich (Gillingham, UK). Red food colourant (Kroma Kolors, Kopykake, Torrance, CA, USA) was purchased from Shesto Limited (Watford, UK). 2-propanol, hydrochloric acid (HCl) (5 M), potassium phosphate, sodium hydroxide (NaOH) (5 M) were purchased from Sigma-Aldrich (Gillingham, UK). All materials were used as received.

### Preparation of formulation

Three formulations with different molecular weights of PEGDA (M_W_ 250 g/mol, 575 g/mol, 700 g/mol) were prepared for volumetric printing, comprising 5% (w/w) paracetamol, 0.025% (w/w) LAP, and a 1:99 (%w/w) ratio of PEGDA: TMAEA (80 wt% in water)). Three equivalent formulations for DLP printing comprising PEGDA (M_W_ 250 g/mol, 575 g/mol, 700 g/mol), TMAEA and LAP, with the addition of red food colourant, were prepared for DLP printing (Table 1). The monomer TMAEA, the photoinitiator LAP, paracetamol, and the colourant (where used) were initially added and stirred for 12 h at room temperature until homogenized. PEGDA was added to each of the formulations and these mixed for a further 12 h. 7 g of resin was prepared for each of the formulations for DLP printing, and 20 g for volumetric printing. All formulations were prepared in amber vials to protect from light exposure and were weighed using an analytical balance. Once the resins were prepared, they were sonicated for 30 min at room temperature using a commercial ultrasonic bath (Ultrasonic Cleaner USC-T, 45 kHz, VWR, Lutterworth, UK). Resins were used immediately afterwards.

**Table 1:** Formulations for DLP and volumetric printing

Formulation	LAP (% w/w)	PEGDA 250 (% w/w)	PEGDA 575 (% w/w)	PEGDA 700 (% w/w)	Paracetamol (% w/w)	TMAEA (% w/w)	Food colourant (% w/w)
DLP250	0.5	0.94	-	-	5	93.06	0.5
DLP575	0.5	-	0.94	-	5	93.06	0.5
DLP700	0.5	-	-	0.94	5	93.06	0.5
VOL250	0.025	0.95	-	-	5	94.03	-
VOL575	0.025	-	0.95	-	5	94.03	-
VOL700	0.025	-	-	0.95	5	94.03	-

### Volumetric printing

**Fig. 1:**
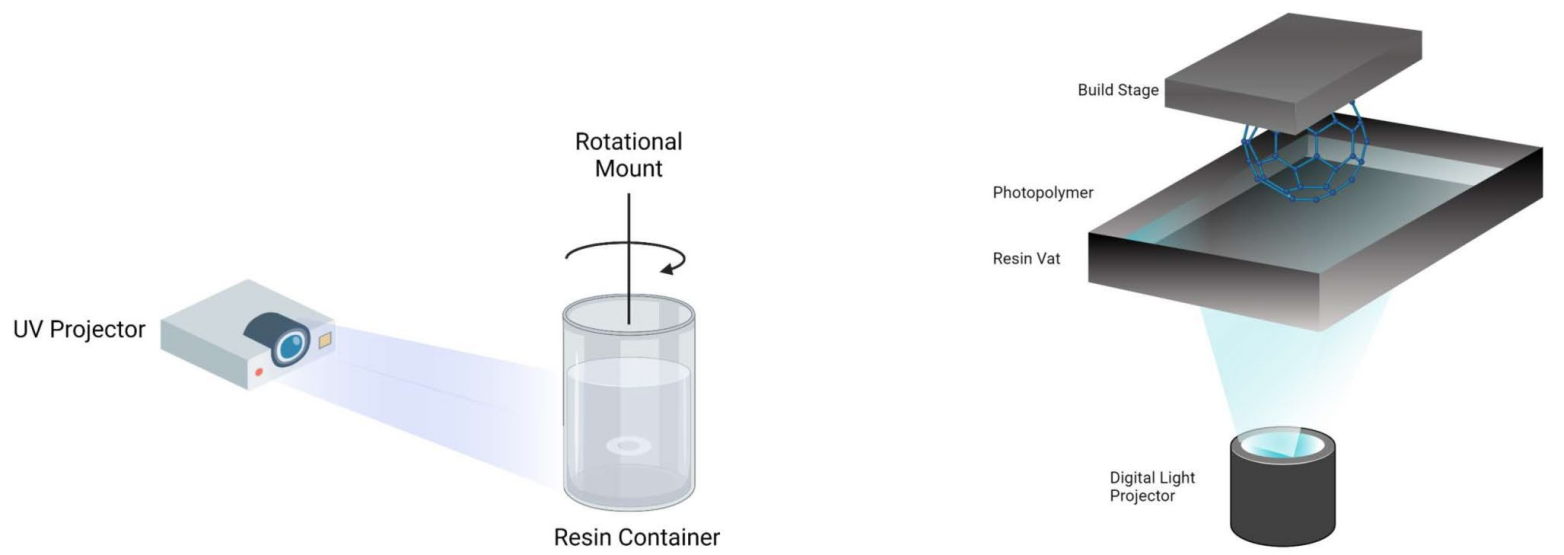
On the left; Volumetric Printer (FabRx Ltd., United Kingdom), On the right; Digital Light Processing (DLP) Printer (Titan2 HR, Kudo3D Inc., Dublin, CA, USA)

The volumetric printer (FabRx Ltd., United Kingdom) consisted of a digital light projector (Wintech DLP6500, San Marcos, California, USA), which emitted UV light at a wavelength of 385 nm in the direction of the rotating resin container (Fig. 1). The cylindrical resin container (2.5 cm diameter x 5 cm height) was suspended by an axis attached to a motor that allowed 360° rotation. The resin container was located at 23 cm from the light source (DLP projector) and at a height of 15 cm from the base.

Tori of dimensions 10 mm diameter x 3 mm height x 3 mm inner diameter were printed. The geometric figures were created using Microsoft Paint (Version 6.3, build 9600), exported in .jpg format, and loaded into software designed by FabRx (London, UK) that controls the printer. The software projects the image of the object in two dimensions on the resin container, which coupled by the rotating motion of the resin container results in the desired 3D structure. Specifically, by projecting an image of two circles, the shape of a torus was obtained. The torus shape was chosen due to its suitability as an oral dosage form. Notably, the high surface area-to-volume ratio facilitates faster drug release compared to conventional cylindrical tablet shapes [23]. Additionally, patients reported high acceptability of torus-shaped oral dosage forms, particularly regarding the perception of ease of swallowing and picking [24].

The photosensitive resin (as prepared in section “Preparation of formulation”) was introduced into the container, which was then attached to the rotary motor via the axis support. The container was carefully positioned at the appropriate height to ensure that the light beam from the projector aligned with the centre of the container. The rotation speed was set to 30 RPM and brightness set to 62.5%. The exposure time used to print each formulation is summarised in Table 2.

**Table 2:** Printing settings for volumetric printing formulations

Formulation	Exposure time (s)
VOL250	6.7
VOL575	7.4
VOL700	7.5

### DLP printing

A DLP printer (Titan2 HR, Kudo3D Inc., Dublin, CA, USA) equipped with an HD DLP projector, which has a visible light source (400–700 nm) was used (Fig. 1). Tori of dimensions 10.2 mm diameter x 3.8 mm height x 3.0 mm inner diameter were printed. 123D Design (Autodesk Inc., Mill Valley, CA, USA) was used to design the torus, which was then exported as a stereolithographic file (.stl) and sliced to layers of a thickness of 25 μm using the Kudo3D Print Job software.

**Fig. 2:**
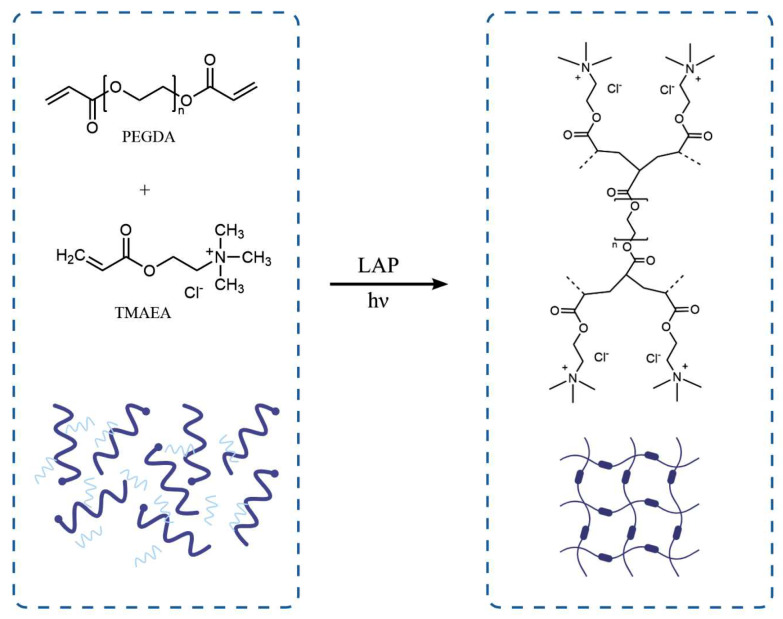
Photopolymerisation reaction of TMAEA and PEGDA monomers to form a hydrogel network. Adapted from [25]

The DLP photosensitive resin (as prepared in the section “Preparation of formulation”) was loaded onto the resin tank and printed. The mutable drug-eluting devices were printed with an exposure time of 30 s per layer and a layer thickness of 25 μm. A total of 152 layers were printed. No supports settings were implemented for the print. Once printing was terminated, the devices were rinsed with isopropyl alcohol for 1 min, placed onto a petri dish and post-cured for 30 min using a commercial curing apparatus (Form Cure, Formlabs Inc., Somerville, MA, USA) which emits 405 nm light. The total print time was approximately 76 min (see Fig. 2).

### Drug loading

#### In device

Devices were immersed in 100 mL of distilled water in volumetric flasks and placed under magnetic stirring for 24 h (*n* = 3). The resulting solutions were diluted 10x with distilled water, and subsequently filtered through a 0.45 μm filter (Merck Millipore Ltd., Cork, Ireland) into HPLC vials. The concentration of drug in each device was then determined using HPLC, as later described in section “High-performance liquid chromatography (HPLC)”.

#### In photosensitive resins

Approximately 0.05 g of each photosensitive resin was extracted after being left stirring overnight to ensure all components had dissolved. These extracts were dissolved in 100 mL of distilled water in a volumetric flask and placed under magnetic stirring for 24 h. Samples were subsequently withdrawn from the volumetric flasks and filtered through a 0.45 μm filter (Merck Millipore Ltd., Cork, Ireland) before HPLC analysis (section “High-performance liquid chromatography (HPLC)”). All measurements were made in triplicates.

### Scanning electron microscopy (SEM)

Devices were cut in half and attached onto a self-adhesive carbon disc mounted on a 25 mm aluminium stub, which was coated with 25 nm of gold using a sputter coater. The stub was then placed into a Quanta 200 FEG Scanning Electron Microscope (FEI, Altrincham, UK) at 5 kV accelerating voltage, using secondary electron detection to obtain the cross-section images of the devices.

### X-ray powder diffraction (XRPD)

Discs (23.0 mm diameter x 1.0 mm height) were printed for each formulation for XRPD analysis, with the same printing settings as used to prepare the respective tori devices. Powdered samples of pure paracetamol were also analysed by XRPD. XRPD patterns were obtained with a Rigaku MiniFlex 600 (Rigaku, Tokyo, Japan) equipped with a Cu Kα X-ray source (λ = 1.5418 Å). The voltage and current applied were 40 kV and 15 mA. Samples were scanned between 3–60° with a step size of 0.02° and a speed of 5°/min.

### Thermal analysis

Differential scanning calorimetry (DSC) measurements were performed with a Q2000 DSC (TA instruments, Waters, LLC, New Castle, DE, USA). Samples were heated at a rate of 10 ºC/min from 0 to 195 ºC, then cooled at 10 ºC/min to 0 ºC and heated again to 195 ºC at 10 ºC/min. Nitrogen was used as a purge gas, with a flow rate of 50.0 mL/min for all experiments. Data were collected with TA Advantage software for Q series (version 2.8.394, TA instruments, Waters LLC, New Castle, DE, USA) and analysed using TA Instruments Universal Analysis 2000. TA aluminium pans and pin-holed hermetic lids (Tzero) were used, with an average sample size of 3.0–5.0 mg.

### Fourier-transform infrared spectroscopy (FTIR)

FTIR was used in this study to examine the spectra both before and after printing. The non-photopolymerized resin was explored, as well as the cured devices and pure paracetamol. The data was collected using a Spectrum 100 FTIR spectrometer (PerkinElmer, Waltham, MA, USA). Each sample was scanned over a range of 4000–650 cm^− 1^ at a resolution of 4 cm^− 1^, with six scans collected.

### In vitro release study

Dissolution profiles for each type of devices were obtained using USP-II apparatus (Model PTWS, Pharmatest, Hainburg, Germany) (*n* = 3). The paddle speed was fixed at 50 rpm and the dissolution medium was maintained at 37 ± 0.5 °C. For the first 2 h, samples were dissolved in 750 mL of 0.1 M HCl. After 2 h, 250 mL of 0.2 M trisodium phosphate solution was added to each dissolution vessel and the pH was adjusted to 6.8 using 5.0 M NaOH solution. 2 mL samples were withdrawn at pre-defined time intervals (5, 10, 20, 30, 45, 60, 90, 120, 150, 180, 240, 300, 360, 420, 480, 720 and 1440 min). Samples were then filtered through a 0.45 μm filter (Merck Millipore Ltd., Ireland) and analysed using HPLC (section“High-performance liquid chromatography (HPLC)”).

Release kinetics were adjusted to zero-order (Eq. 1), first-order (Eq. 2), Korsmeyer Peppas (Eq. 4), and Weibull (Eq. 5) models. The R^2^ values of each model were obtained and compared for all formulations to elucidate the underlying release mechanism.

*Zero-order*

1 $$\:{C}_{t}\:=\:{C}_{0}\:+\:{K}_{0}\:t$$

Where C_t_ represents the amount of drug released at time t, C_0_ represents the initial concentration of drug released, K_0_ represents the zero-order constant.

*First-order*

2 $$\:\frac{dC}{dt}=\:-{K}_{1}t$$

Where K is the first order rate constant expressed in units of time. After integration, the equation can be expressed as

3 $$\:Log\:\left({C}_{t}\right)=\:-\:log\:\left({C}_{0}\right)-Kt/2.303$$

Where C_0_ is the initial concentration of drug released, and C_t_ is the concentration of drug at time t.

*Korsmeyer-Peppas*

4 $$\:\frac{Mt}{M\alpha\:}=\:K{t}^{n}$$

Where M_t_/M_α_ is a fraction of drug released at time t, K is the rate constant, and n is the release exponent which indicates mechanistic release behaviours.

*Weibull*

5 $$\:M={M}_{0}\left[1-{e}^{-\frac{{(t-T)}^{b}}{a}}\right]$$

Or

6 $$\:M=1-{e}^{{-(t-T)}^{b/a}}$$

Where M is the amount of drug release as a function of time *t*. M_0_ represents the total amount of drug released, T is the lag time of drug release, which in this case T = 0. Parameter *a* defines the timescale of the process, and *b* characterises the shape and type of curve.

### High-performance liquid chromatography (HPLC)

A Hewlett Packard 1260II Series HPLC system equipped with an online degasser, quaternary pump, column heater, autosampler and UV/Vis detector, was used. 20 µL of the samples were injected into an Eclipse plus C18 5 μm column, 4.6 × 150 mm (Zorbax, Agilent technologies, Cheshire, UK). Solution compounds were separated by a mobile phase consisting of 15% of methanol and 85% of water with a 1 mL/min flow rate. The process was carried on with a temperature of 40 °C and eluents were then screened with a 247 nm light source.

### Gravimetric sorption assay

Gravimetric sorption assays were performed to examine the degree of swelling and uptake kinetics of the hydrogel devices. Experiments were conducted in both purified distilled water and simulated gastric fluid (Biorelevant FaSSGF) to assess device swelling behaviour under physiologically relevant gastric conditions.

For each formulation (*n* = 3), individual devices were pre-weighed and immersed in 300 mL of the respective test medium. At predetermined time intervals (30, 60, 90, 120, 150, 180, 240, 300, 360, 420, 480, 720, and 1440 min), devices were removed, carefully blotted dry, and re-weighed. The degree of swelling was calculated using the following equation:

$$\:Swelling\:Ratio\:\left(\%\right)=\:\frac{{W}_{t}-{W}_{0}}{{W}_{0}}\times100$$

where *W*_*t*_ is the weight (g) of the hydrogel device at time *t* and *W*_*0*_ is the initial weight (g) of the hydrogel device, and swelling ratio is presented as a percentage.

## Results & discussion

### Volumetric printing

Volumetric printing was successfully adapted to formulate and create torus shaped drug-eluting devices that demonstrate controlled mechanical and dimensional changes over time. TMAEA was used as the primary matrix monomer, which enables supramolecular interactions that can be reversibly disrupted to enable stimuli response. PEGDA was used as a crosslinker to hold the polymerised supramolecular polymers together, preventing dissolution of the printout. Paracetamol was used as a model drug, and LAP was selected as the photoinitiator due to its strong absorption at the wavelength 385 nm, and high biocompatibility in bioprinting applications [18, 19]. However, the relatively hydrophobic nature of PEGDA [13] was observed to create micelles in the TMAEA-based resin, leading to a turbid, immiscible resin composition at high PEGDA ratios.

The turbidity of the resin is a critical factor that determines the printing accuracy and resolution of the print in volumetric printing, as the light patterns used to photopolymerise the resin must not be distorted or attenuated in order to yield geometrically accurate prints [26]. High turbidity causes scattering of light emitted by the projector and causes photopolymerisation of undesired areas. Thus, the use of highly turbid resins would result in inaccuracies in printing. Taking this into account, a formulation of 1:99 (PEGDA: TMAEA) was ultimately selected. This formulation balances the threshold between a low turbidity and high optical transparency for accurate prints, while retaining a sufficient amount of PEGDA crosslinkers to achieve the desired swelling properties of the hydrogel. To further reduce the turbidity of the resins, sonication was employed. Sonication has been utilised in various fields to decrease the size of micelles and induce structural changes [27–29]. On this basis, sonicating the resin allows for changes in micelle formation, size, and structure, which resulted in lower visible turbidity, and thus reduced light scattering. Devices prepared with the sonication method were observed to have better resolution, with smooth surfaces and accurate dimensions.

**Fig. 3:**
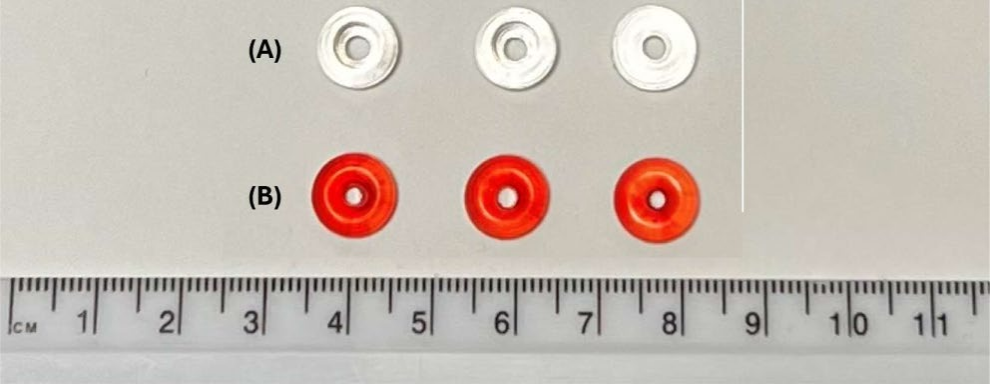
Photograph of devices (**A**) VOL250, VOL575, VOL700 (from left to right), and (**B**) DLP250, DLP575, DLP700 (from left to right)

The cumulative light dose threshold that induces polymerization differs for each formulation due to the varying average molecular weight (Mn) of PEGDA used. PEGDA with different Mw possess different reactivity, and therefore photopolymerization occurs at different rates. As such, the exposure times of the formulations were adjusted accordingly, with the formulation containing PEGDA 700 requiring 7.5s, PEGDA 575 requiring 7.4s, and PEGDA 250 requiring the least amount of time, 6.7s. These printing parameters resulted in the highest resolution prints, with high definition and details. The volumetric printed devices had smooth surfaces, defined curvatures, and were transparent (Fig. 3). Moreover, by virtue of the volumetric printing process—printing in three dimensions simultaneously rather than layer-by-layer addition—the devices were shown to have a well-defined circular torus shape.

### DLP printing

The DLP formulations contained a higher concentration of LAP (0.5%) than volumetric printing (0.025%) due to the different light sources employed by each technology. Initial trials utilizing formulations equivalent to volumetric printing, without colorants, yielded poor resolution in DLP-printed devices. This was attributed to the increased per-layer exposure time and multiple exposures inherent to the layer-by-layer DLP process, resulting in photopolymerization extending beyond the intended geometry. Therefore, food colorant was incorporated as a photoabsorber to control light penetration, alter the photopolymerization threshold, and ensure high print resolution.

**Fig. 4:**
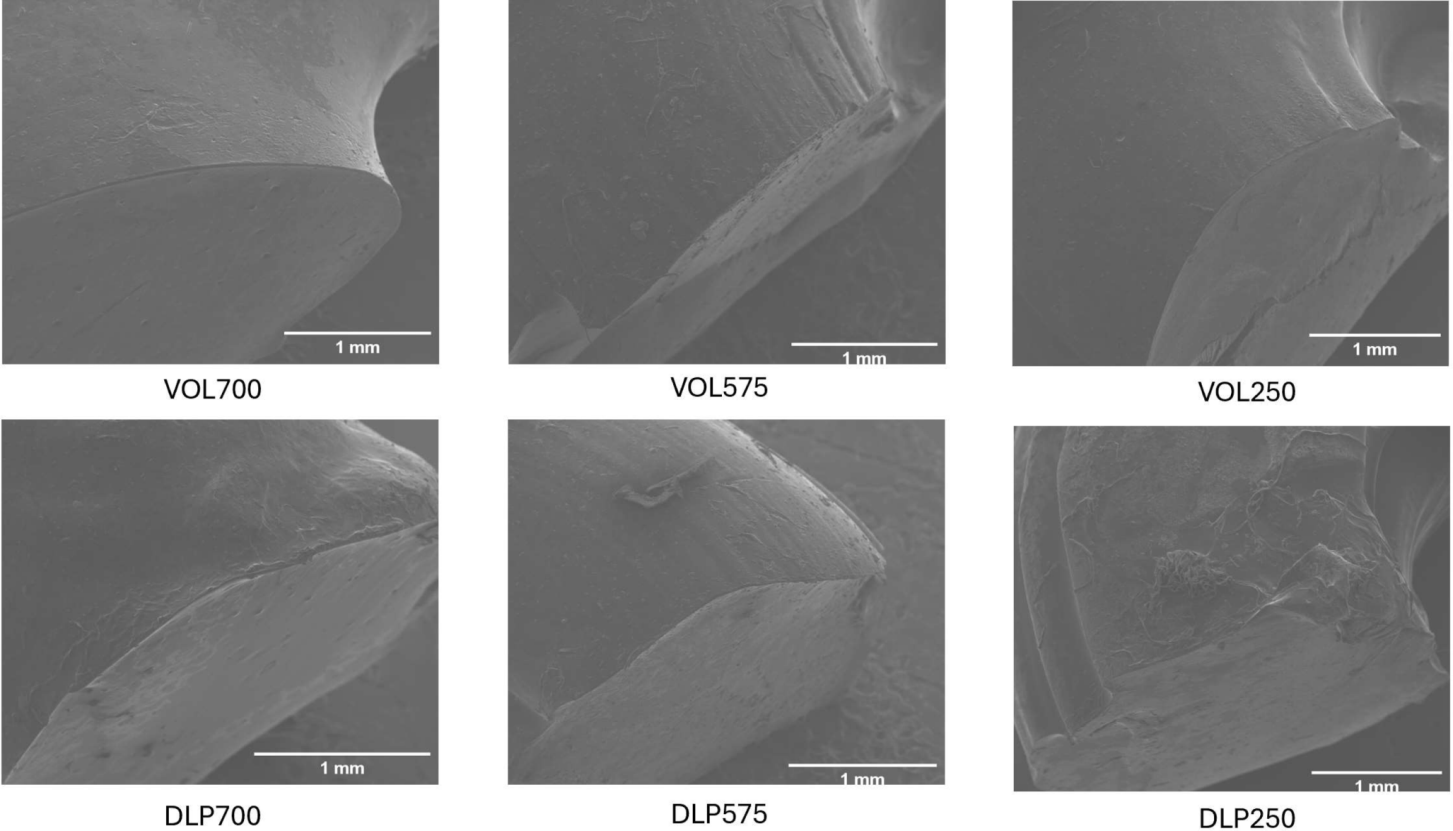
SEM images of torus device cross-sections: VOL700, VOL575, VOL250 (top, from left to right), DLP 700, DLP575, DLP250 (bottom, from left to right)

The DLP devices showed characteristic horizontal layers on the surface created by the layer-by-layer process (Fig. 4). Further, by virtue of this process, DLP-printed tori exhibited noticeably flat bottom layers compared to their volumetric counterparts, which displayed smooth, well-defined curves. This difference arises from DLP’s layer-by-layer approach requiring a flat surface to adhere to the build plate. For shapes like the torus, achieving similarly curved geometries requires the additional fabrication of temporary supports. While acceptable in other industries, this approach is undesirable in pharmaceutics due to material waste (including unused drug) and a manually intensive removal process, both of which may be costly [30].

Moreover, DLP printing showed clear limitations in printing speeds in comparison to volumetric printing. Based on the size of the printing platform and projection display size, a total of six devices could be printed simultaneously, with a total print time of approximately 76 min, resulting in a per device printing time of 12.7 min. While scaling up the printing platform and projection display could increase the number of simultaneous prints, it would also significantly increase excess drug-containing materials needed due to the minimum material requirement to cover the projection area. Volumetric printing overcomes these limitations by simultaneously printing the entire object in 3D, requiring no supports. Coupled with rapid printing speeds, volumetric printing is not restrained by minimum material requirements by virtue of the vessel design and fabrication process described in Section “Volumetric printing”. This unique capability facilitates the creation of complex, curved geometries with minimal drug waste, offering a significant advantage in the pharmaceutical sector.

### Characterisation of resins and devices

The drug loading of paracetamol in all formulations was found to be slightly lower than the theoretical load (5% w/w) (Table 3). Significant difference was found between the drug loading of DLP and volumetric devices, with volumetric devices exhibiting a higher mean drug loading across all formulations (*p* < 0.05). DLP and volumetric printed formulations containing PEGDA 575 presented the highest relative drug loading, while PEGDA 700 containing formulations showed the lowest drug loading. Drug loading in the photosensitive resins also was determined using HPLC with acceptable variability within ± 2.49% RSD.

**Table 3:** Device dimensions, weight, drug loadings of DLP and VOL devices, and resin drug loadings

Formulation	Diameter (mm) (*n =* 9)	Height (mm) (*n =* 9)	Weight (mg) (*n =* 9)	Resin loading (%, w/w ± SD) (*n =* 3)	Device loading (%, w/w ± SD) (*n =* 3)
VOL250	9.98 ± 0.12	3.63 ± 0.22	294.70 ± 8.13	5.056 ± 0.026	4.90 ± 0.02
VOL575	9.90 ± 0.29	3.60 ± 0.25	277.44 ± 12.84	5.046 ± 0.002	4.95 ± 0.04
VOL700	9.96 ± 0.13	3.55 ± 0.19	275.00 ± 10.98	4.959 ± 0.009	4.77 ± 0.01
DLP250	9.91 ± 0.08	3.48 ± 0.06	270.47 ± 6.90	4.940 ± 0.028	4.47 ± 0.05
DLP575	9.93 ± 0.12	3.50 ± 0.08	277.26 ± 7.47	5.042 ± 0.103	4.62 ± 0.02
DLP700	9.90 ± 0.06	3.49 ± 0.06	281.22 ± 6.49	4.928 ± 0.123	4.47 ± 0.02

All formulations were able to be printed with consistent dimensions. The diameter of devices ranged by no more than ± 2.93% RSD, height by no more than ± 6.94% RSD, and weight was determined to have a range of no more than ± 4.63% RSD.

Scanning electron microscopy (SEM) images confirmed the internal structure of each formulation, showing a cross-sectional view of the torus devices. The devices printed using the DLP displayed an evident layer by layer structure, while devices fabricated using volumetric printing exhibited a uniform and layer-free structure. This is due to the volumetric-printed devices being fabricated directly in three-dimensions simultaneously, as opposed to the repeated two-dimensional layering processes seen in other additive manufacturing technologies, including DLP printing. Devices printed using both the DLP and volumetric printer display a uniform cross-section, void of evident crystalline particles, indicating the homogeneity of the formulation and the absence of crystallised drugs.

**Fig. 5:**
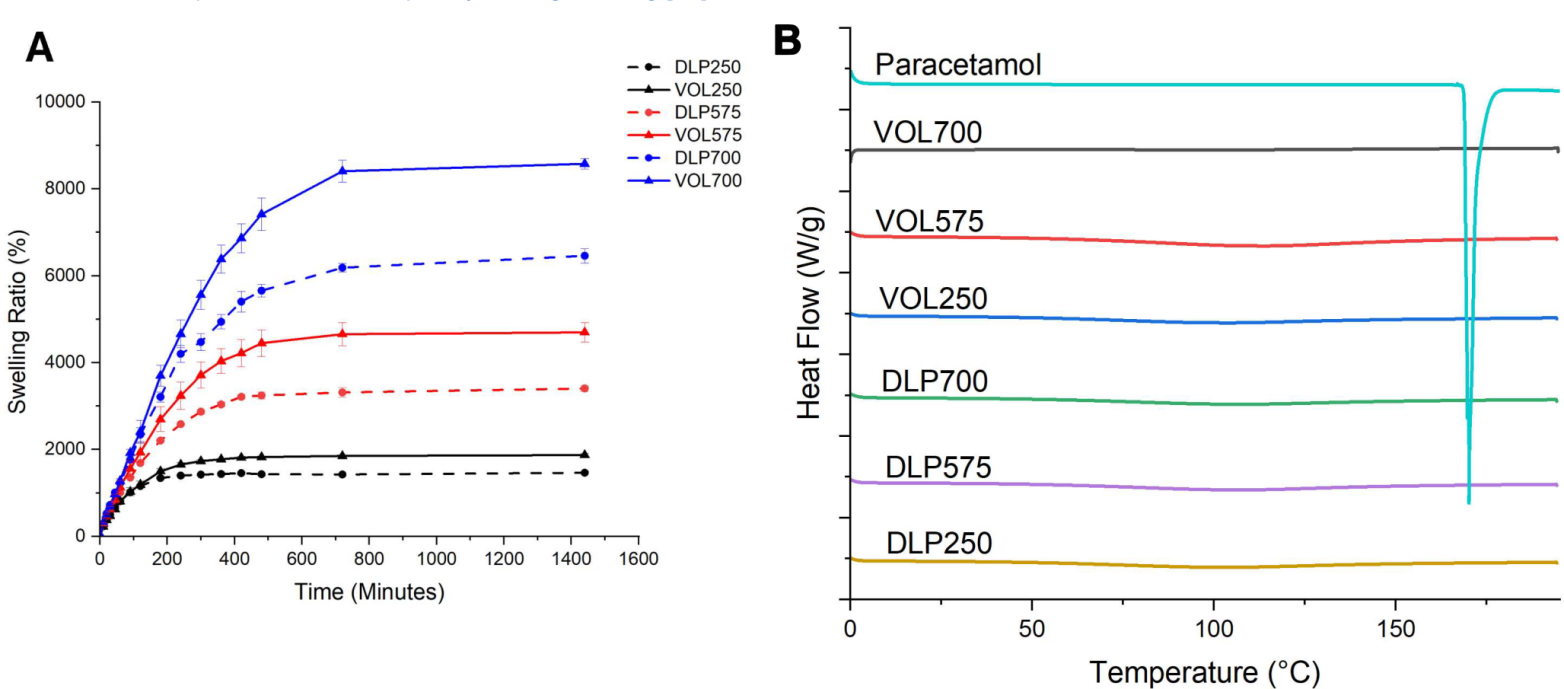
DSC thermograms of paracetamol, DLP devices, and VOL devices. Exo up

Thermal analysis using DSC (Fig. 5) was employed to assess the physical state of paracetamol within the devices. Pure paracetamol has a well-defined melting point of 169 ºC, characterised by a sharp endothermic peak. The absence of a peak at the melting point of paracetamol in the device thermograms indicate complete dissolution of the drug in the liquid resin formulations, confirming that paracetamol is present as a molecular dispersion in the solid device. All devices exhibited broad endothermic peaks over a wide temperature range during the initial DSC heating cycle. This is likely due to evaporation of atmospheric moisture sorbed by the highly hygroscopic hydrogels, which can retain substantial volumes of water within their cross-linked network.

**Fig. 6:**
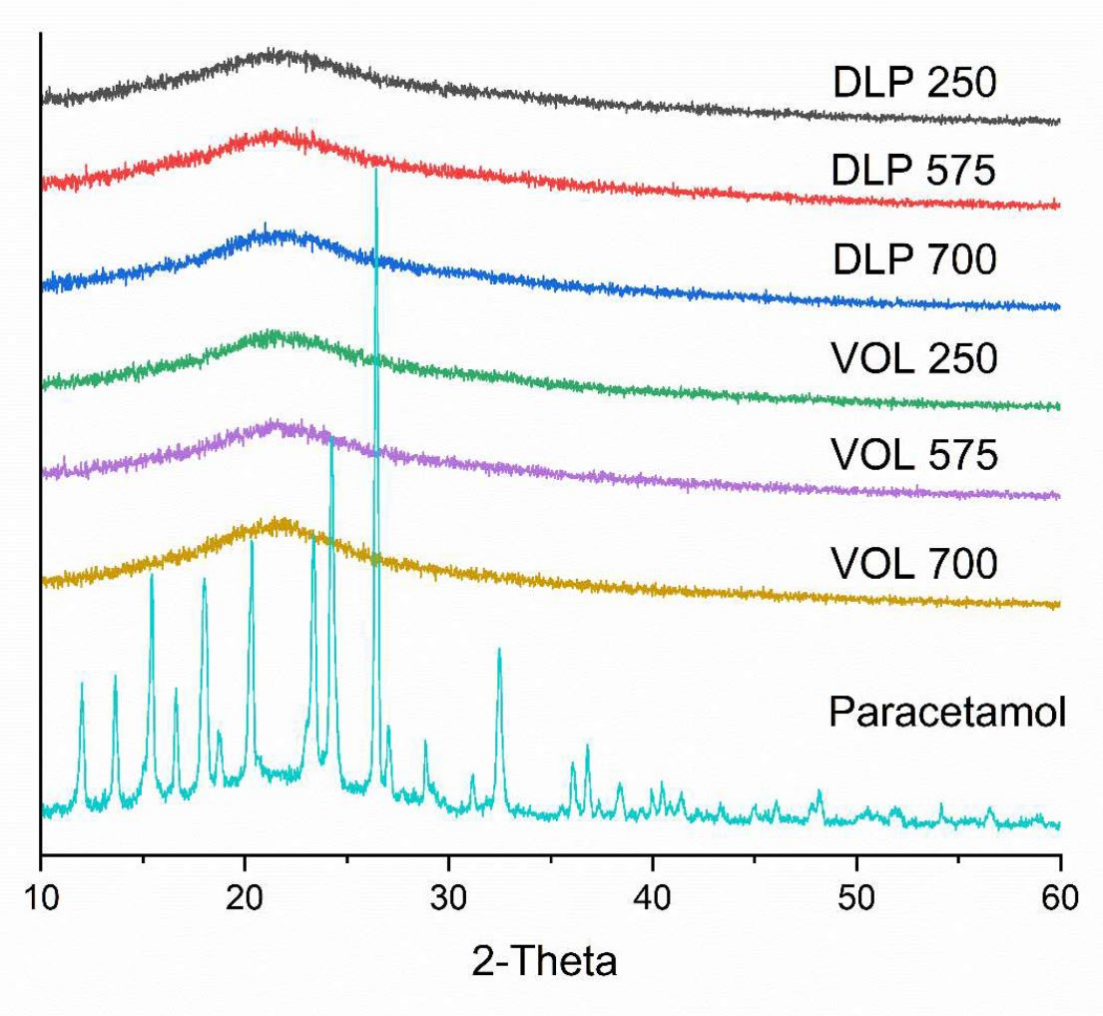
XRPD patterns of the printed devices and paracetamol

XRPD (Fig. 6) was used to confirm the findings of DSC. XRPD patterns of pure paracetamol showed a series of intense Bragg reflections, clearly indicating the crystalline nature of the paracetamol. In contrast, the prepared devices show no distinct Bragg reflections. Instead, only a broad halo is present in each of the patterns, consistent with amorphous solid dispersions having been formed.

**Fig. 7:**
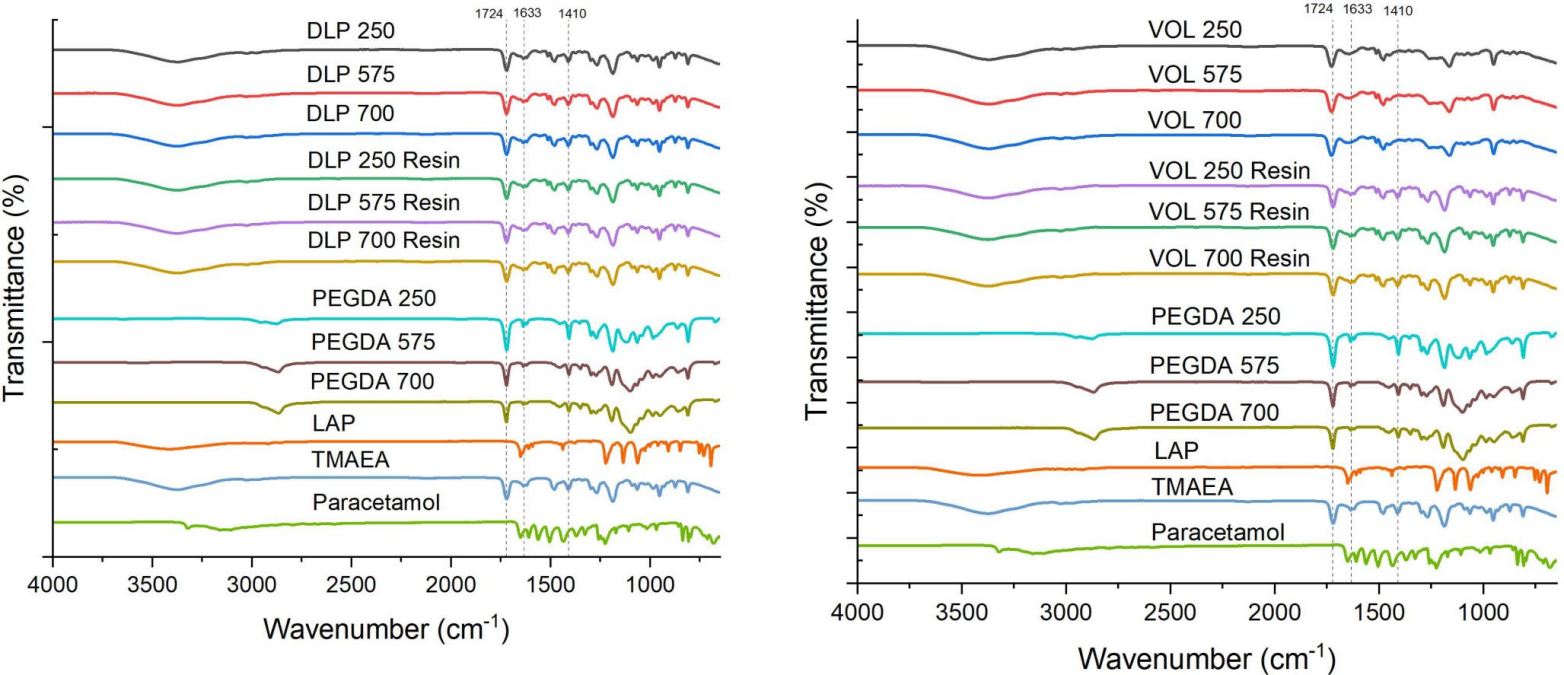
FTIR analysis of DLP devices of varying molecular weights (top), and volumetric devices (bottom) along with their respective resins and formulation components

The FTIR spectrum (Fig. 7) of pure paracetamol exhibited distinctive peaks at 1610 cm-1 (C = C stretching), 3321 cm-1 (N-H amide stretching), 3200 cm-1 (O-H stretching) and 1654 cm-1 (C = O stretching) [30, 31].

For comparison, all the resin components were also measured. Analysis of the TMAEA FT-IR spectrum revealed characteristic peaks at 1724 cm-1 (ester C = O stretch), corresponding to the acrylate group, and at 1633.

cm-1 (C = C stretch), corresponding to the alkene group. Following polymerisation, as observed from the FT-IR of the devices (e.g. VOL 250, 575, 700), attenuation in the alkene C = C intensity at 1633 cm-1 was observed, signifying the conversion of the alkene double bonds into single bonds (C-C) during the formation of the hydrogel polymer network through acrylate radical polymerisation.

**Fig. 8:**
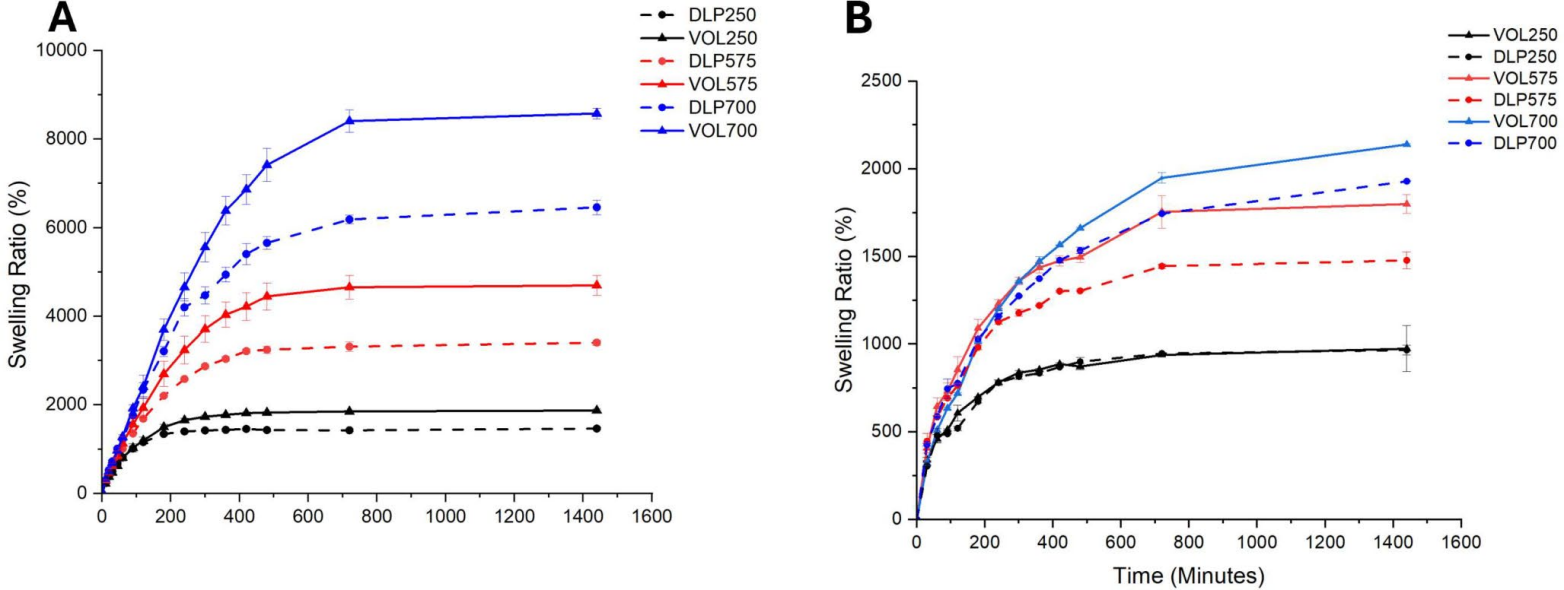
Gravimetric swelling kinetics of volumetric (straight lines, *n* = 3) and DLP (dotted lines, *n* = 3) devices created with different crosslinker molecular weights (250, 575, 700). (**A**) water (**B**) Simulated Gastric Fluid

The degree of swelling was measured gravimetrically, as the dimensions and solution uptake directly correlate to the changes in mass of the devices (Fig. 8). Both DLP and volumetric devices exhibited a high degree of swelling in distilled water, with all prints surpassing a 1400% increase in mass. The observed high swelling degree aligns well with previous studies demonstrating an inverse relationship between swelling ratio and effective crosslinking density [31, 32]. This relationship arises because reducing the crosslinker concentration leads to lower crosslinking density, consequently resulting in higher swelling ratios [33, 34]. High crosslinking densities would create tighter hydrogel structures, limiting the degree of penetration of absorbed liquid into the hydrogel mesh structure [35]. Consistent with this, previous studies have shown that reducing the relative concentration of PEGDA in hydrogels induces higher degrees of swelling [25, 36]. Comparing devices derived from equivalent formulations (i.e., same PEGDA Mn used), the volumetric devices were observed to have significantly higher swelling ratios compared to DLP devices (*p* < 0.05). This may potentially be attributed to the inclusion of red food colourant in the DLP printed devices leading to higher crosslinking densities, thus reducing swelling.

It was observed that the degree of swelling differs depending on the average molecular weight of PEGDA used in the formulation, with larger molecular weight PEGDAs leading to a higher degree of swelling. This observation is in line with the hypothesis that the shorter monomer chains of PEGDA 250 cause the hydrogel to expand to a lesser degree than longer PEGDA crosslinker chains, due to the smaller structural mesh size limiting the amount of water sorbed and stored in the hydrogel matrix. Studies investigating the effect of PEGDA molecular weights on the properties of hydrogels of equivalent polymer concentration have demonstrated that hydrogels containing lower molecular weight PEGDA exhibit a lower swelling ratio compared to their counterparts with higher molecular weight PEGDA at equivalent compositions [37].This was attributed to the higher relative content of acrylate groups in the lower molecular weight macromers leading to a higher crosslinking density. Consequently, the resulting hydrogel network possesses a smaller mesh size, which in effect decreases water absorption. A similar phenomenon is observed in this work.

However, the swelling behaviour of the devices in simulated gastric fluid (SGF) diverged significantly from those in distilled water. While the relative swelling capacity between formulations remained consistent across both media, a marked reduction in the absolute degree of swelling was observed in SGF for all formulations (Fig. 8). Notably, VOL700 devices, despite exhibiting the highest swelling in SGF, reached a maximum of only 2139%, a substantial decrease compared to their maximum swelling degree of 8580% in distilled water.

**Fig. 9:**
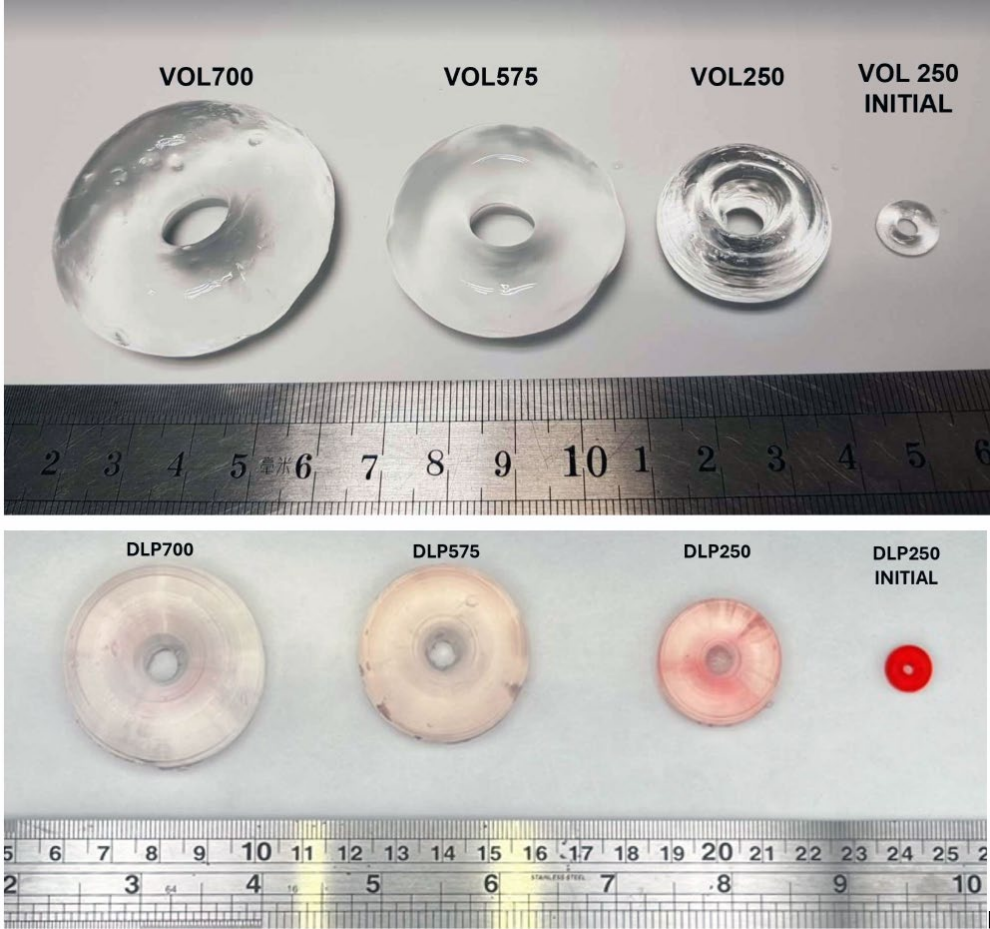
Comparison of volumetric (top) and DLP device (bottom) dimensions before and after 24 h in distilled water

This pronounced impact of low pH on swelling may be attributed to alterations in the electrostatic repulsion within the hydrogel network. Electrostatic repulsion between charged groups on the polymer chain is known to contribute to swelling through mechanisms such as chain expansion and increased osmotic pressure [38]. The positively charged quaternary ammonium group in TMAEA plays a crucial role in these electrostatic interactions. In acidic conditions, increased interactions between positively charged hydrogen ions (H+) and the chloride counterions associated with the quaternary ammonium group may lead to charge screening, reducing electrostatic repulsion and subsequently limiting swelling [39] (see Fig. 9).

**Fig. 10:**
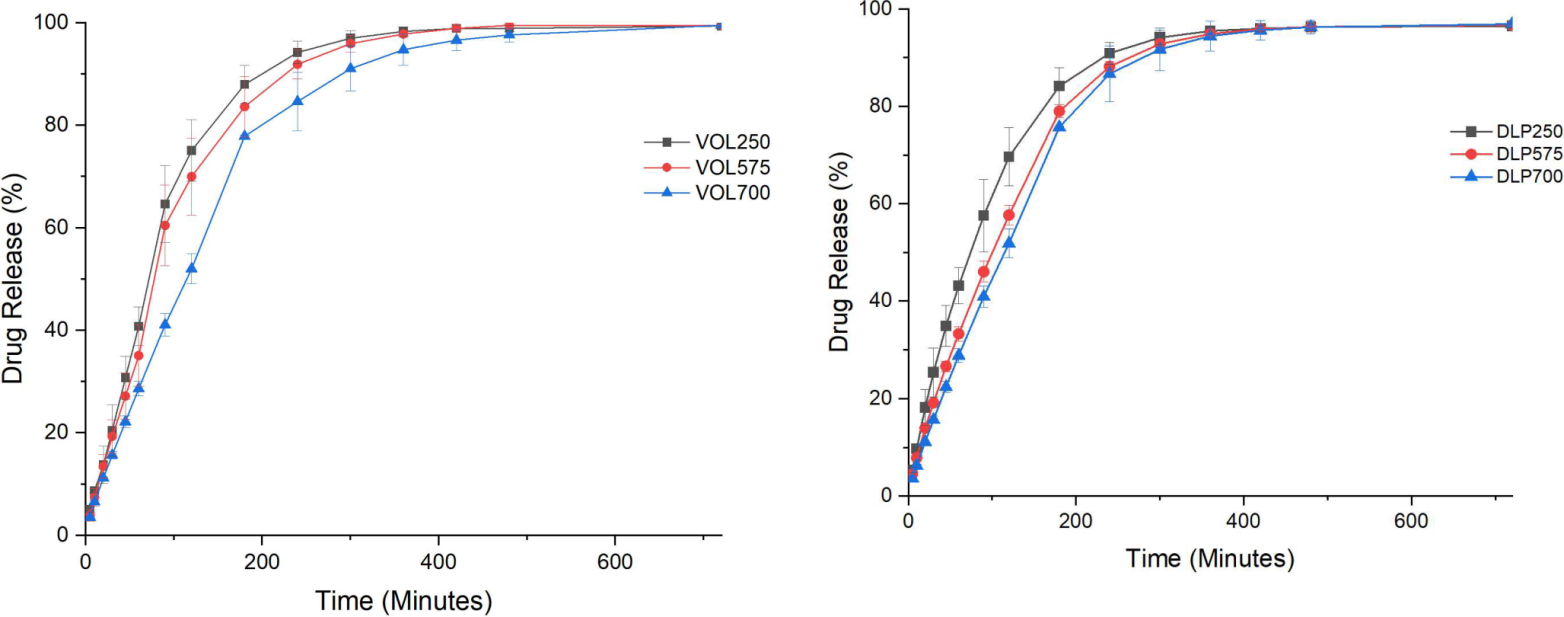
Drug release kinetics of volumetric (top) and DLP devices (bottom)

The drug release profiles of DLP and volumetric devices were determined via in vitro dissolution tests and are shown in Fig. 10. The results indicated that paracetamol release commenced during the gastric phase and release profiles remained unaffected by pH changes after 2 h. All devices had increased in size after 24 h, in accordance with what was observed in the gravimetric sorption experiments (Fig. 8). 90% drug release for the printed formulations was achieved within 4 h for DLP250, VOL250, and VOL575 devices, and within 5 h for DLP 575, DLP700 and VOL700 devices. Drug release profiles for DLP and volumetric devices were compared using FDA F1 F2 tests, which validated the similarity of the release profiles.

Drug release seemed to correlate to the speed of expansion and the water uptake equilibrium of the devices. This can be attributed to the swelling process causing the polymer network to become less dense, which facilitates passive diffusion of paracetamol molecules out of the matrix [25, 40]. Both VOL250 and VOL575 devices were shown to approach their maximum water retention capacity at a faster rate than VOL700 devices.

As hydrogels swell and approach a state of equilibrium, the polymer network allows for more efficient drug diffusion out of the network, in turn leading to faster drug release [40, 41]. VOL250 and VOL575 devices were found to approach this state of equilibrium at a faster rate than VOL700 devices, leading to faster drug release seen in the former compared to the latter. This is comparable to previous studies showing slower rates of drug release observed in more densely crosslinked polymer networks [13]. This also applies to DLP devices; however, due to the lower degree of swelling, the difference between the formulations was less pronounced compared to the volumetric devices. It must be noted that the model drug, paracetamol, readily dissolves upon contact with fluids entering the hydrogel matrix due to its high solubility, resulting in rapid drug diffusion. With a less soluble drug, the impact of the different formulations on release kinetics may be more pronounced. Further studies are needed to confirm this hypothesis, which may demonstrate the platform’s potential for tailoring drug release kinetics based on individual drug properties.

**Table 4:** R^2^ results for various drug release models for mutable devices

Formulation	Zero-order	First-order	Korsmeyer-peppas	Weibull
DLP250	0.4194	0.9875	0.9445	0.9853
DLP575	0.8486	0.9879	0.9775	0.9996
DLP700	0.8798	0.9907	0.9571	0.9664
VOL250	0.7755	0.9935	0.9194	0.9754
VOL575	0.8115	0.9896	0.9341	0.9538
VOL700	0.8845	0.9874	0.9595	0.9808

Various release kinetics models were fitted onto drug release profiles to determine the underlying release mechanism of the printed mutable devices (Table 4). The first-order model performed the best, demonstrated to be the better fit across all formulations except DLP575, where the Weibull model proved superior. The first-order and Weibull models suggest that drug release is primarily governed by diffusion through the porous hydrogel matrix, with release rate proportional to the remaining drug concentration. This aligns with the observed decrease in drug release over time.

## Conclusion

Volumetric printing was utilized to create mutable drug delivery devices within a short time frame (6.7 to 7.5 s per device), a significant time saving compared to Digital Light Processing printing’s 12.7 min per device. These drug delivery devices were shown to possess high water sorption properties that allow them to swell up to 85-times the initial weight and increase dimensionally upon exposure to fluids, which may prove useful in various drug delivery systems. The devices printed using the volumetric printer exhibited similar physicochemical properties to the DLP printed devices, and all existed as amorphous solid dispersions. The volumetric formulations demonstrated a greater degree of swelling and water sorption than the DLP systems, and similar drug release profiles. Notably, the degree and rate of swelling were correlated with the PEGDA molecular weights. This direct comparative study underscores the promising capability of volumetric printing in the rapid manufacture of stimuli-responsive drug delivery devices. Its speed, potential for curved geometries, and layerless approach offer distinct advantages over established methods, expanding the repertoire of pharmaceutical printing technologies.

## Electronic supplementary material

Below is the link to the electronic supplementary material.

Supplementary Material 1

## Data Availability

The datasets generated during and/or analysed during the current study are available from the corresponding author on reasonable request.
