# Supplementary material for: Fabrication of 3D printed mutable drug delivery devices: a comparative study of volumetric and digital light processing printing
Source: Drug Deliv Transl Res. 2024 Aug 23;15(5):1595–608. doi: 10.1007/s13346-024-01697-5 (PMC11968558; doi:10.1007/s13346-024-01697-5)

**Supplementary Data:**

Volumetric Printing Projection:

The following static image was projected onto the volumetric printer apparatus using UV rays to photopolymerise photosensitive resins.


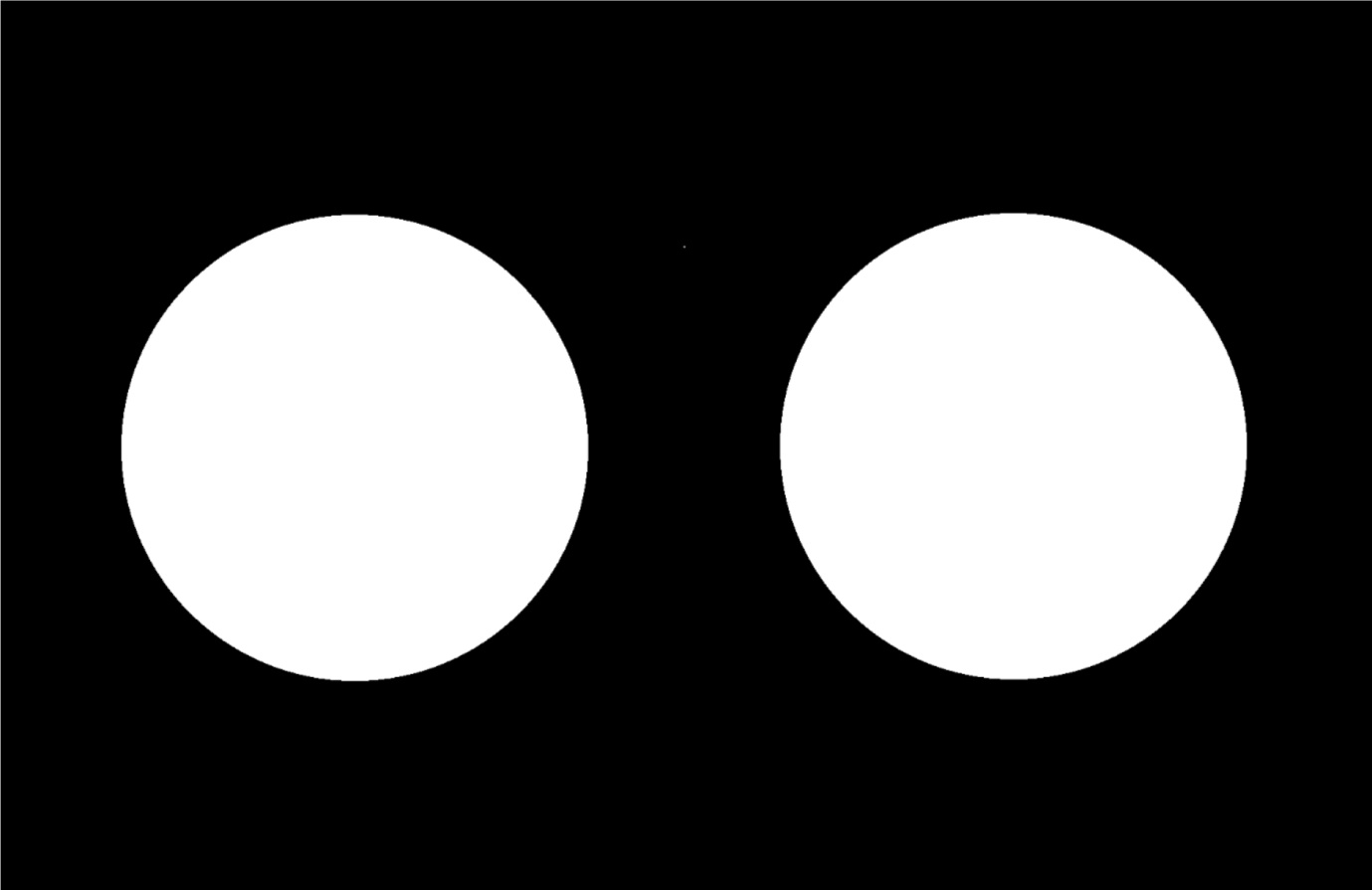


Gravimetric Swelling Studies in Simulated Gastric Media:

The following images show the dimensional increase of devices post-24 hour swelling in FASSGF. (Top: Volumetric Printed devices, ordered from left to right: VOL700, VOL575, VOL250, VOL250 pre-swelling, Bottom: DLP Printed devices, ordered from left to right: DLP700, DLP575, DLP250, DLP 250 pre-swelling)


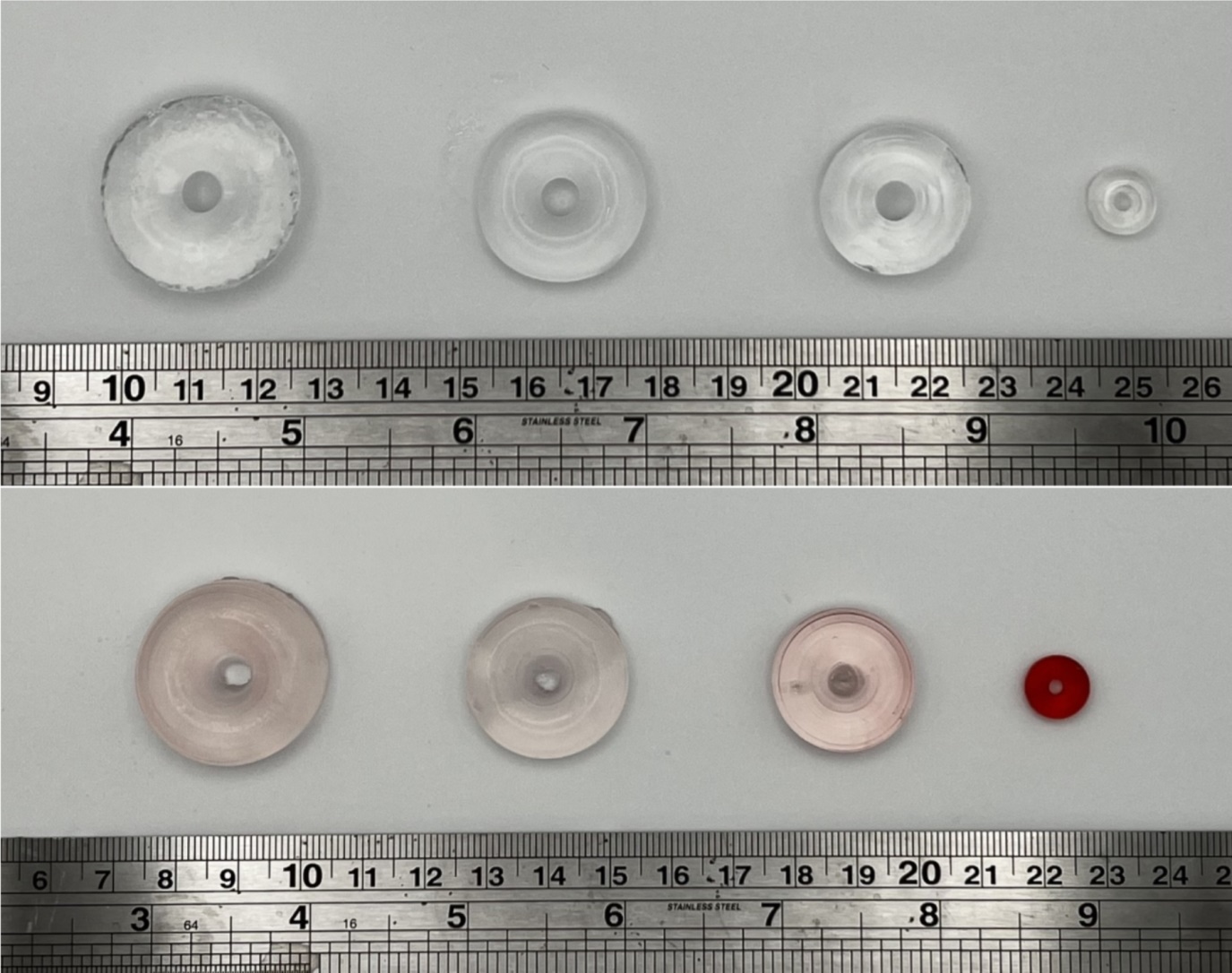

Supplement: Supplementary file 1 — Supplementary Material 1 [file 13346_2024_1697_MOESM1_ESM.docx]
